# Tight Junction Modulating Bioprobes for Drug Delivery System to the Brain: A Review

**DOI:** 10.3390/pharmaceutics12121236

**Published:** 2020-12-19

**Authors:** Keisuke Tachibana, Yumi Iwashita, Erika Wakayama, Itsuki Nishino, Taiki Nishikaji, Masuo Kondoh

**Affiliations:** 1 Graduate School of Pharmaceutical Sciences, Osaka University, Osaka 565-0871, Japan; iwashita-y@phs.osaka-u.ac.jp; 2 School of Pharmaceutical Sciences, Osaka University, Osaka 565-0871, Japan; wakayama-e@phs.osaka-u.ac.jp (E.W.); nishino-i@phs.osaka-u.ac.jp (I.N.); nishikaji-t@phs.osaka-u.ac.jp (T.N.)

**Keywords:** blood-brain barrier (BBB), tight junction (TJ), claudin, angulin, angubindin-1, antibody, *Clostridium perfringens* enterotoxin (CPE), *Clostridium perfringens* iota-toxin (Ia)

## Abstract

The blood-brain barrier (BBB), which is composed of endothelial cells, pericytes, astrocytes, and neurons, separates the brain extracellular fluid from the circulating blood, and maintains the homeostasis of the central nervous system (CNS). The BBB endothelial cells have well-developed tight junctions (TJs) and express specific polarized transport systems to tightly control the paracellular movements of solutes, ions, and water. There are two types of TJs: bicellular TJs (bTJs), which is a structure at the contact of two cells, and tricellular TJs (tTJs), which is a structure at the contact of three cells. Claudin-5 and angulin-1 are important components of bTJs and tTJs in the brain, respectively. Here, we review TJ-modulating bioprobes that enable drug delivery to the brain across the BBB, focusing on claudin-5 and angulin-1.

## 1. Introduction

**Figure 1:**
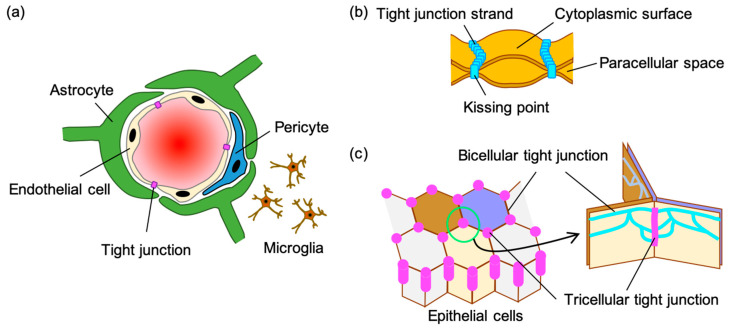
Illustration of the blood-brain barrier and tight junction (TJ). (**a**) Components of the blood-brain barrier. The blood-brain barrier is formed by vascular endothelial cells, pericytes, astrocytes, and microglial cells. The vascular endothelial cells form tight junctions. (**b**) Schematic structure model of a tight junction strand. Tight junctions tightly associated laterally to each other form a paired tight junction strand (kissing point). The intercellular space is completely obliterated at the kissing point [8]. (**c**) Structural model of a tight junction. Bicellular TJs and tricellular TJs, which are a structure at the contact of two cells and three cells, respectively [9].

The existence of the blood-brain barrier (BBB) has been suggested by Paul Ehrlich’s experiments with aniline dyes in the late 19th century [1]. The BBB separates the brain’s extracellular fluid from the circulating blood, plays a role in protecting the brain from pathogens and other substances, and maintains the homeostasis of the central nervous system (CNS). The BBB consists of four types of cells: endothelial cells, pericytes, astrocyte end-feet, and microglial cells (Figure 1a). Epithelium acts as a barrier separating the inside of the body from the outside environment, and epithelial and endothelial cells form tight junctions (TJs) by sealing the paracellular spaces [2]. TJs control the diffusion of ions and solutes across the paracellular spaces to maintain homeostasis and to prevent the absorption of drugs into the body and the delivery of drugs into tissues (Figure 1b) [3]. BBB endothelial cells form TJs and express specific polarized transport systems to tightly control paracellular movements of solutes, ions, and water. Based on successful CNS drugs, small molecules that fit the Lipinski’s “Rule of Five”, comprising molecular weight, lipophilicity, polar surface area, hydrogen bonding, and charge, are favorable for BBB penetration. Furthermore, the efflux transporters in the BBB, such as P-glycoprotein (P-gp), excrete drugs from the brain and are major obstacles to drug penetration into the brain. Therefore, P-gp substrates are not desirable for CNS-targeted drug discovery [4,5,6]. More than 98% of small-molecule drugs fail to penetrate the brain. Thus, many researchers in the field of drug discovery and development are trying to develop BBB drug delivery technologies for the treatment of CNS diseases [7].

To date, the following technologies have been developed to deliver drugs into the brain based on the functions of the BBB: receptor-mediated transcytosis, transferrin receptor and insulin receptor; solute carrier-mediated transcytosis, L-type amino acid transporter 1 (LAT1) and glucose transporter type 1 (GLUT1); and drug efflux transporters, P-glycoprotein (P-gp) [10]. These drug delivery strategies were developed to target the transcellular pathway. BBB disruption with mannitol, a hyperosmolar agent, is already used clinically for drug delivery via paracellular transport into the CNS. Although the osmotic opening of the BBB with mannitol may allow the delivery of antineoplastic drugs to patients with brain tumors, the interendothelial TJs are estimated to spread in a width of approximately 20 nm, and this uncontrolled opening of TJs poses a risk of undesired molecules (such as toxins) entering the brain [11,12]. Consequently, techniques for modulating size-selective BBB openings to enable safe drug absorption are being developed worldwide. Here, we review and discuss the safety of TJ modulators for drug delivery to the brain.

## 2. Tight Junction of the Blood-Brain Barrier

**Figure 2:**
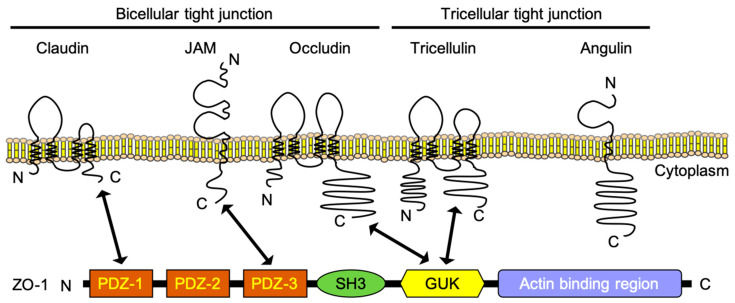
Schematic structures of tight junction proteins [8,14,15]. Arrows indicate the interactions between represented tight junction proteins and scaffold protein zonula occludens (ZO)-1. JAM, junctional adherence molecule. PDZ, postsynaptic density-95, discs-large, ZO-1. SH3, Src Homology-3. GUK, guanylate kinase.

The formation of TJs in the BBB requires transmembrane proteins, namely the claudin family, angulin family, TJ-associated myelin and lymphocyte (MAL) and related proteins for the vesicle trafficking membrane link (MARVEL) protein (TAMP) family, junctional adherence molecule (JAM) family, and zonula occludens (ZO) family proteins, which are scaffold proteins of those membrane proteins (Figure 2). There are two types of TJs: bicellular TJs (bTJs) and tricellular TJs (tTJs), which are structures at the contact of two cells and three cells, respectively (Figure 1c). Claudins and occludin, a TAMP family protein, are essential factors in the formation of bTJs [8], and the TAMP family protein tricellulin and angulin family proteins are key proteins in the formation of tTJs [13,14].

### 2.1. Claudin-5 and Other Claudins

Claudins are tetra-transmembrane proteins and, since claudin-1 and claudin-2 were identified in 1998, 27 members of this family have been discovered in mammals [16,17]. Claudins are composed of an intracellular N- and C-terminus, two extracellular loops, a large fist extracellular segment (ECS1), a shorter second extracellular segment (ECS2), and an intracellular loop [18]. Found at the C-terminus of claudins, there is a postsynaptic density-95, discs-large, ZO-1 (PDZ) domain-binding motif for binding to a scaffold protein such as ZO-1, and a C-terminal containing a transmembrane domain required for TJ localization (Figure 2) [19]. Claudins have the property of interacting with other claudin family members on the same (cis-interaction) and opposite (trans-interaction) cell membranes, thereby generating a TJ strand [20]. Claudins have two extracellular regions that are essential for the interaction between claudin family members, and the differences in the amino acid sequences of these regions among claudins cause differences in the abilities of claudin-based TJs [21]. Additionally, as each claudin family member has a different tissue expression profile, the TJ of each tissue shows a unique strength and permeability. Found at the BBB, multiple subtypes of claudins are expressed and, in particular, claudin-5 is predominantly expressed compared with the other subtypes [22].

Claudin-5 was identified in 1999 through a similarity search with a sequence that has been reported as a common deletion within velo-cardio-facial syndrome [23]. Claudin-5, like other members of the claudin family, has a tetra-transmembrane domain and consists of 218 amino acids, with a molecular weight of 23 kDa. Claudin-5 is widely expressed in various organs [24,25], and it is expressed in endothelial cells [26,27]. Introduction of claudin-5 into rat brain capillary endothelial cells (TR-BBB) resulted in significantly lower permeability of the TR-BBB/claudin-5 monolayer [22]. Thus, claudin-5 has been shown to exhibit a particularly strong interaction ability among claudin family members, which might be related to the strong TJ properties of the BBB [28].

Although claudin-5-deficient mice died within 10 h of birth, morphological examination of the brain capillaries of claudin-5-deficient mouse embryos showed no differences against those of the wild-type and those with no hemorrhage or edema in the brain. Additionally, no major abnormalities were detected in various hematoxylin–eosin-stained tissues. Tracer experiments in claudin-5-knockout mice revealed that small molecules of 1 kDa or less, such as Hoechst stain H33258 (562 Da) and gadolinium-diethylenetriamine-*N,N,N′,N″,N″*-pentaacetic acid (742 Da), passed through the BBB, but microperoxidase (1.9 kDa) and tetramethylrhodamine-conjugated dextran (10 kDa) did not [29]. Furthermore, a size-selective, transient BBB opening was observed in siRNA-induced claudin-5 knockdown mice. The permeation of small molecules of up to 742 Da, but not molecules of 4.4 kDa, from the brain microvessels was observed for up to 48 h after the injection of claudin-5 siRNA in mice, with no significant adverse effects. Furthermore, the administration of neuropeptide thyrotropin-releasing hormone (360 Da) from the tail vein to the mice after the injection of claudin-5 siRNA inhibited the permeation for up to 5-times longer than that observed in non-target control mice [30].

During an analysis using a human brain capillary endothelial cell line (BCEC) induced to express C-terminally tagged murine claudin-5-yellow fluorescent protein (YFP) by doxycycline (hCMEC/D3-mCLDN5-YFP), an in vitro model of human BBB showed that the murine claudin-5-YFP-transduced hCMEC/D3 cells exhibited a significant increase in the transepithelial/transendothelial electrical resistance (TEER) levels, indicating the integrity of TJ dynamics in cell culture models, and a decrease in paracellular permeability compared to those in parental hCMEC/D3 cells (211 Ω cm^2^ for hCMEC/D3-mCLDN5-YFP cells versus 117 Ω cm^2^ for hCMEC/D3 cells). However, the barrier properties of hCMEC/D3-mCLDN5-YFP cells were still lower than those of porcine BCECs that generally show a high TEER level [31,32]. These results suggest that besides claudin-5, other proteins are involved in the formation of the BBB. Indeed, glucocorticoid hydrocortisone, which increases the TEER level and improves the BBB function, induced the barrier function in hCMEC/D3 cells (324 Ω cm^2^) by upregulating claudin-5 and occludin [33]. 

Recently, a gene expression analysis of frozen brain tissue obtained by laser capture microdissection revealed that the expression of claudin-1, -5, -11, -12, -25, and -27 was higher than that of occludin as a TJ marker in human cortical capillaries [34]. Seen in capillaries microdissected from mice, the transcript level of claudin-5, -11, -12, and -25 was higher than that of occludin, and the protein level of claudin-5 was the highest among all claudins in the purified mouse brain capillaries [34]. Claudin-11 contributed to the formation of strong TJs, and the expression of claudin-11 was downregulated in the brain and spinal cord capillaries of experimental autoimmune encephalomyelitis (EAE) model mice, widely used as an animal model of CNS inflammation multiple sclerosis, and in patients with multiple sclerosis [34,35]. Although functional analyses of CNS myelin using claudin-11 knockout mice have been performed [36,37], there is no report of a BBB permeability analysis, necessitating further research to elucidate the in vivo contribution of claudin-11 at the BBB. Regarding claudin-12-lacZ knock-in mice, claudin-12 was expressed at low levels in brain endothelial cells compared to that in other cell types in the body; this suggests that claudin-12 is not required for the BBB TJ function [38]. Although claudin-12 null mice showed reduced calcium permeability in the proximal tubule, the function of the BBB TJ has not yet been reported [39]. The knock down of claudin-25, also known as claudin domain containing 1, in the mouse brain endothelial cell line bEND.3 did not affect TEER, but resulted in unstructured TJ strands and a reduced protoplasmic mesh number [34]. Furthermore, Ohnishi et al., reported that claudin-25 knockdown increased the permeability of human brain endothelial cells to fluorescence-conjugated dextran (4 kDa) [40]. There are no reports of claudin-25 knockout mice, and research on the functions of claudin-25 in in vivo claudin-25 knockout mice is expected in the future.

The above findings suggest that in claudin-5 knockout mice, the presence of other claudin-based TJs could maintain the structural integrity of endothelial cells in the brain and prevent bleeding [29]. Quite the reverse, persistent suppression of claudin-5 in adult doxycycline-inducible claudin-5 knockdown mice resulted in seizures and the mice died 3–4 weeks after claudin-5 suppression [41]. This suggests that long-term loss of the BBB barrier function, even by molecules less than 1 kDa, compromises the safety of the BBB opening [42]. Thus, claudin-5 plays an important role in the barrier properties of the BBB, and the lack of adverse effects such as brain hemorrhage, edema, and behavioral changes due to transient claudin-5 knockdown is important and advantageous from the perspective of drug delivery to the brain.

### 2.2. Occludin

Occludin is the first identified TJ protein; this TAMP family protein is a tetra-transmembrane protein with a molecular weight of 6–6.5 kDa (Figure 2) [43]. Although occludin is an important component of TJs, occludin itself, unlike claudins, cannot form a TJ strand. However, occludin, upon interaction with claudins, can form a complex TJ strand and enhance barrier function [44]. Although occludin-deficient mice show no phenotype of TJ formation and strand morphology, histological abnormalities such as calcification around the vascular endothelial cells in the brain were observed [45].

Regarding EAE model mice, the expression of both claudin-5 and occludin proteins decreased with an increase in the expression of vascular endothelial growth factor A (VEGF-A), and this, in turn, resulted in the BBB opening [46]. Intriguingly, although the injection of claudin-5 or occludin siRNA injection into mice did not increase the BBB permeability of the biotinylated dextran (3 kDa), the co-administration of claudin-5 and occludin siRNAs allowed molecules of up to 3–4 kDa to diffuse across the BBB via the paracellular pathway, but not molecules of 10 kDa. Additionally, in the Tg2576 mouse model of Alzheimer’s disease that found overexpression of a mutant form of the amyloid precursor led to an increased brain amyloid-β (Aβ) level and impaired cognate functions, chronic administration of siRNAs targeting claudin-5 and occludin significantly increased the plasma Aβ(1-40) (4.3 kDa) levels and decreased the brain Aβ(1-40) levels. Subsequently, the cognitive function of mice was enhanced. These chronic administrations of these siRNAs did not show any evident toxicity in the peripheral organs [47]. These results suggest that the co-suppression of both occludin and claudin-5 can modulate size-selective BBB permeability.

### 2.3. Tricellulin and Angulin-1

A tTJ is formed where three cells meet. Tricellulin is the first discovered protein that constructs tTJs and is an essential protein for the formation of tTJs and bTJs [9]. Tricellulin is a tetra-transmembrane protein consisting of 558 amino acids, with a molecular weight of 63 kDa. The C-terminus region of tricellulin is necessary for binding to the scaffold protein, ZO-1 (Figure 2). Tricellulin, occludin, and MarvelD3 are highly homologous and called TAMP. Tricellulin plays an important role in the barrier function, which depends on the localization of tricellulin. Tricellulin localized at tTJs and bTJs is involved in the permeability of macromolecules (4–10 kDa), ions, and large solutes, respectively [48]. However, mice lacking tricellulin do not show an abnormal phenotype associated with the BBB [49]. 

Angulin is a single-transmembrane protein with immunoglobulin-like extracellular domains and an intracellular domain that functions as a tricellulin recruiter, recruiting tricellulin to the intersection of the three cells to form a tTJ (Figure 2) [50]. The angulin family has three subtypes. Angulin-1, which also is known as a lipolysis-stimulated lipoprotein receptor (LSR) [51], is localized to endothelial cells in the brain [34]. The knockout of angulin-1 decreased the barrier function, but there were no abnormalities in the ultrastructure of the brain blood vessels in the angulin-1-deficient mice. Although angulin-1-deficient mice died before embryonic day 15.5, leakage of the small molecule Sulfo-NHS-biotin (446 Da) into the brain was observed, but not large endogenous proteins, such as albumin (69 kDa), antibodies (160 kDa), and fibrinogen (52 kDa). Leakage of small molecules into the brain was not observed in adult angulin-1 heterozygous knockout mice; thus, one copy of angulin-1 is sufficient to maintain the BBB permeability. These findings indicate that angulin-1 might regulate the size-selective permeability of the BBB. Additionally, in BBB-disrupted experimental models, such as the EAE model and a middle cerebral artery occlusion (MCAO) model of stroke, the expression of angulin-1 in inflammatory and leaky lesions of blood vessels was significantly downregulated [52]. These results indicate that angulin-1 plays an important role in the barrier function of the BBB.

The transcript levels of both tricellulin and angulin-1 were increased in CNS endothelial cells compared to those in peripheral endothelial cells [53,54]. However, tricellulin is distributed ubiquitously in several tissues, and the effect of the molecular size of molecules on their permeability depends on the localization of tricellulin in bTJs or tTJs [48,49]. Quite the opposite, angulin-1 is specifically expressed in tTJs of vascular endothelial cells that form the BBB [50,53]; therefore, angulin-1 is a promising target in CNS drug delivery.

### 2.4. Junctional Adherence Molecules (JAM-A)

JAM family proteins, first reported in 1998, are type I transmembrane proteins belonging to the immunoglobulin superfamily that have two immunoglobulin-like loops, with a molecular weight of 40 kDa (Figure 2) [55]. JAMs are dimerized on the same cell membrane and form tetramers by interacting with a dimer between opposite cell membranes [56]. 

JAM-A is highly expressed in vascular endothelial cells in the brain. JAMs can interact with lymphocyte function-associated antigen-1, which plays an important role in the extravasation of lymphocytes. The administration of a JAM-A-blocking monoclonal antibody (BV11) to a mouse model of acute cytokine-induced meningitis with increased BBB permeability reduced the number of leukocytes that penetrated the brain [57]. During a rat cortical cold injury model, the expression of JAM-A significantly decreased in the lesion site after brain damage [58]. It was reported that knockdown of JAM-A in human dermal microvascular endothelial cells caused claudin-5 and ZO-1 to disappear from TJs [59]. Recently, Kakogiannos et al. revealed that JAM-A promotes the expression of the transcription factor CCAAT/enhancer-binding protein α (C/EBPα), and C/EBPα induces the expression of claudin-5. Additionally, the expression of claudin-5 in the vasculature of various tissues, including the brain, was significantly reduced in JAM-A-deficient mice, which lost size-selective barrier function as claudin-5-deficient mice [60]. However, JAM-A-deficient mice did not show a lethal phenotype like claudin-5-deficient mice [61].

### 2.5. Zonula Occludens (ZO)-1

The ZO family belongs to the membrane-associated guanylate kinase homologs (MAGUK) family and is composed of three members. ZO-1, identified in 1986, is a peripheral membrane phosphoprotein with a molecular weight of 225 kDa [62,63]. ZO family proteins are scaffold proteins that bind to claudins, occludin and tricellulin, and JAMs via the PDZ1 domain, GUK domain, and PDZ3 domain, respectively (Figure 2) [15]. ZO-1 and ZO-2 have many overlapping functions and are expressed on endothelial cells [64]. Regarding ZO-1 and ZO-2 double knockout cells, the TJ structure disappeared and even 150 kDa macromolecules could diffuse throughout the paracellular space [65]. Concerning human dermal microvascular endothelial cells, ZO-1 appears to mainly contribute to the formation of TJs, and knockdown of ZO-1 alone causes claudin-5 and JAM-A to disappear from TJs [59]. ZO-1-deficient mice showed vascular development defects associated with the mislocalization of endothelial junctional adhesion molecules, and died before embryonic day 11.5 [66]. The role of ZO-2 in the BBB is still unclear.

## 3. Tight Junction Modulators for Drug-Delivery Systems (DDSs)

Outlined in the previous section, claudin-5 and angulin-1 are abundantly expressed in brain endothelial cells, and mice lacking claudin-5 or angulin-1 have a size-selective loosened BBB [29,52]. Therefore, both claudin-5 and angulin-1 are considered candidate targets for drug delivery to the BBB. Regarding the discovery of drugs targeting membrane proteins, molecules that bind to extracellular regions, such as antibodies, are the first choice. However, in the case of claudins, owing to their small extracellular region (the first extracellular loop consists of approximately 50 amino acids and the second extracellular loop consists of approximately 25 amino acids) and high protein sequence homology among various species [67], it was difficult to develop claudin binders containing antibodies against the extracellular region [68]. Hence, studies have initially been conducted using toxin fragments that target TJs [69].

### 3.1. Claudin Binders

#### 3.1.1. Fragment of Bacterial Toxins

**Figure 3:**
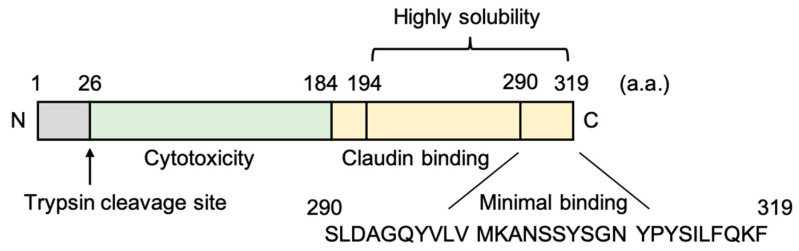
Structure of *Clostridium perfringens* enterotoxin [71]. The GenBank protein accession number is ADE93025.

*Clostridium perfringens* enterotoxin (CPE), a 35-kDa polypeptide consisting of 319 amino acids, causes food poisoning in humans [70]. CPE has two functional regions, the N-terminal region is cytotoxic, and the C-terminal region (C-CPE184; 184–319 amino acids, ~15 kDa) binds to its receptors claudin-3 and claudin-4 with high affinity (Figure 3) [71,72,73]. The C-terminal region of CPE (C-CPE) does not show cytotoxicity and modulates the function of the epithelial TJ barrier by binding to its receptors [72]. Although C-CPE binds not only to claudins-3 and -4 but also to claudins-6, -7, -8, -9, -14, and -19, C-CPE cannot bind to claudin-5 [74,75,76]. The deletion construct of 10 amino acids of the N-terminal of C-CPE184 exhibits a high solubility and affinity for claudins (C-CPE194; 194–319 amino acids) and modulates the TJ barrier [77,78].

**Figure 4:**
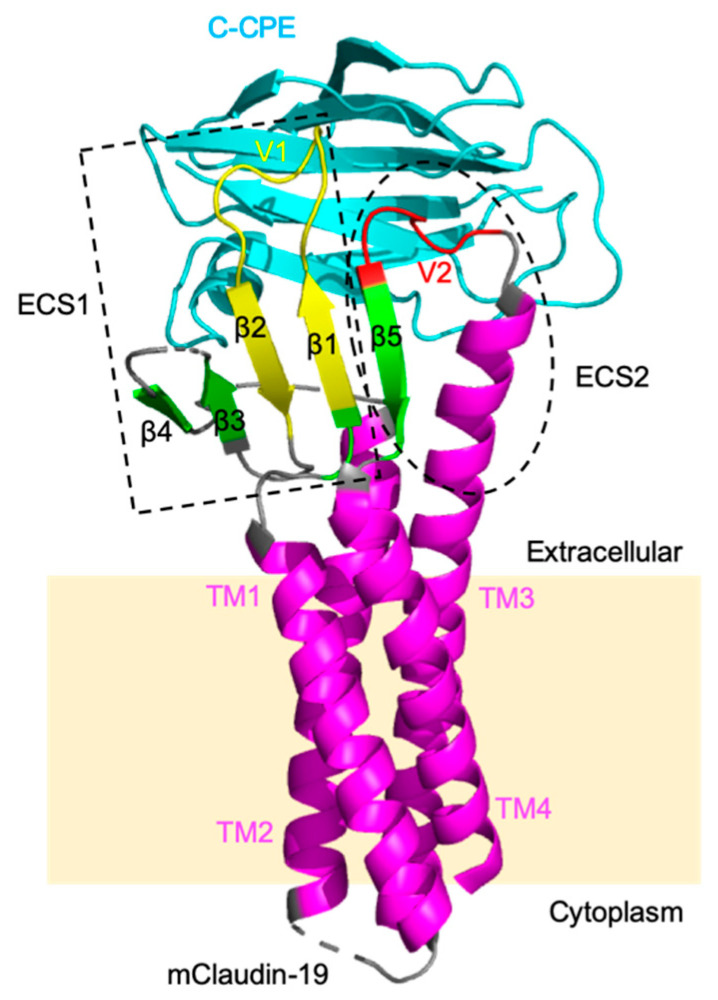
Structure of mouse claudin-19 in complex with C-terminal *Clostridium perfringens* enterotoxin (C-CPE) (Protein Data Bank (PDB): 3X29) [80]. Mouse claudin-19 has four transmembrane helices (TM1–TM4) (pink) and two extracellular segments (ECS)1 (dashed square) and ECS2 (dashed circle). The variable regions of (V)1 and V2 are displayed in yellow and red, respectively. C-CPE is colored blue. C-CPE interacts with ECS1 and ECS2 in mouse claudin-19.

The crystal structures revealed that mammalian claudins morphologically resemble the left hand of humans; claudins have four transmembrane helices (TM1–TM4) corresponding to the forearm and two extracellular segments (ECS1 and ECS2) containing a β-sheet of five β-strands (β1–β5) corresponding to the four fingers and thumb (Figure 4) [79,80,81,82,83]. The extracellular helix (ECH) at the end of ECS1 interacts hydrophobically with the extracellular region of TM3 of the neighboring claudin within the same membrane (cis-interactions). The extracellular variable regions V1, the loop between β1 and β2 of ECS1, and V2, the loop between TM3 and β5 of ECS2, are required for head-to-head adhesion in opposing lateral cell membranes (trans-interactions). The crystal structures of the mouse claudin-19, human claudin-4, or human claudin-9 in complex with C-CPE revealed that C-CPE interacts with the two extracellular regions of claudin, ECS1 and ECS2 (Figure 4). It has been reported that C-CPE induces conformational changes the ECH in the extracellular region of claudins and disrupts the lateral assembly of claudins [80,81,82,83].

Previously, we and others generated C-CPE mutants that can bind to claudin-5. C-CPE m19 (S304A/S305P/S307R/N309H/S313H) was obtained by screening claudin binders from a C-CPE mutant-displaying phage library using claudin-displaying budded baculovirus. It could recognize various claudins (claudins-1, -2, -4, and -5) [84,85]. C-CPE mt (Y306W/S313H) was designed based on the crystal structure of claudins. The binding specificity of C-CPE mt to claudin-5 was increased over wild-type C-CPE; moreover, this mutant bound to claudin-1 and showed weakened binding to some claudins (claudins-3, -4, -6 to -9) [86,87]. C-CPE (N218Q/Y306W/S313H) also was designed by structure-based mutagenesis, and it bound more strongly to claudin-5 and more weakly to claudin-1 and -4 than wild-type C-CPE [88]. 

These claudin-5-binding C-CPE mutants enhanced the solute permeation of in vitro BBB models [87,88]. The treatment of in vitro BBB models, mouse cell line cerebEND, primary rat brain endothelial cells with rat astrocytes (pRBMEC/AST), and primary porcine brain endothelial cells (pPBMEC), with C-CPE mt and C-CPE (N218Q/Y306W/S313H) decreased TEER in a concentration-dependent and reversible manner without cytotoxicity. These C-CPE mutants increased the permeability of carboxyfluorescein (375 Da), but not that of fluorescence-conjugated dextran (4 kDa). Freeze fracture electron microscopy revealed that the C-CPE mt treatment did not substantially affect the overall structure or total breakdown of the TJs [88]. During a nonhuman primate BBB model, primary cynomolgus monkey brain microvasculature endothelial cells with rat pericytes and astrocytes, C-CPE mt time-dependently reduced TEER and increased the BBB permeability of the fluorescence-conjugated dextran (4 kDa) without cytotoxicity [87].

During an in vivo experiment, C-CPE mt co-localized with claudin-5, allowing the passage of Texas red (3 kDa) and rhodamine B-dextran (10 kDa) across the larval zebrafish BBB. C-CPE mt affected the permeability of the BBB for up to 3 h after injection, but this BBB permeability was unaffected 4 h after injection. This finding suggests that C-CPE variants can reversibly modulate the BBB permeability and they are suitable for drug delivery to the brain in zebrafish [89]. To contrast, C-CPE mt could not deliver a 16-mer gapmer antisense oligonucleotide (approximately 5.3 kDa) to the brain of mice. C57BL/6 mice receiving C-CPE mt showed no abnormal behavior or liver or kidney dysfunction, as assessed by histological and serum chemistry analyses. Additionally, mice treated with Evans blue solution following C-CPE mt showed no extravasation of Evans blue, which is known to bind to serum albumin and act as a macromolecule (approximately 70 kDa) in the blood, into the brain [90]. Although in vivo drug delivery to the brain by C-CPE mutants requires further investigation, including a safety assessment, these findings indicate that C-CPEs are useful tools for drug delivery to the BBB.

#### 3.1.2. Antibodies against Claudin-5

Due to the small extracellular region of claudins and their high degree of homology among species, immunization of mice and rats with claudins is difficult [67,68,91,92]. Our group successfully generated monoclonal antibodies against the extracellular regions of human claudin-5 using the claudin-5 proteoliposome or the eukaryotic expression plasmid encoding human claudin-5 (DNA immunization method) [87,93]. A flow cytometry-based cellular binding assay revealed that most of the generated monoclonal antibodies can specifically bind to human and cynomolgus monkey claudin-5, but not to mouse claudin-5. These antibodies decreased TEER in canine epithelial MDCKII cells expressing human or cynomolgus monkey claudin-5, but not in mouse claudin-5-expressing MDCKII cells (Table 1). The decrease in TEER caused by the antibodies occurred without cell injury, and it was recovered by approximately 24 h after the antibodies were washed out. Furthermore, the ability of the antibodies to modulate the TJ barrier was evaluated using an in vitro BBB model, primary cynomolgus monkey brain microvasculature endothelial cells, with rat pericytes and astrocytes. The results revealed a significant decrease in the TEER and an increase in the penetration of small-molecule fluorescent tracers (both sodium fluorescein (376 Da) and fluorescence-conjugated dextran (4 kDa)) through the intercellular space. Furthermore, anti-claudin-5 antibodies altered the subcellular localization of claudin-5 from the cell membrane to the membrane and cytoplasm in cynomolgus monkey brain endothelial cells [87,93].

Claudin-5 also is expressed at low to moderate levels in the intestinal epithelium, but the contribution of claudin-5 to the barrier function of these epithelial cells is still unknown [24]. Interestingly, an analysis using cells transfected with claudin-5 revealed that claudin-5 increased TJ permeability in the moderately high resistance human intestinal epithelial cell line, Caco-2, and did not affect the paracellular barrier in the high resistance Madin–Darby canine kidney cell line, MDCK-C7 [94]. Additionally, the antibodies against claudin-5 did not affect the TEER of human intestinal epithelial cell line T-84 that expresses claudin-5 [87]. Thus, except in the vascular endothelial cell barrier, where claudin-5 is a main component of TJ, the contribution of claudin-5 to the barrier function is considered to be low. This is similar to the finding that claudin family member-deficient mice did not show barrier dysfunction in tissues expressing claudins but showed a lack of barrier function only in specific tissues (see above). Therefore, the use of the anti-claudin-5 antibody may regulate the function of the BBB without affecting the TJ in other epithelial tissues.

Regarding claudin-5, three amino acids in the extracellular domains, D68 in the extracellular loop domain (ECL) 1, T75 in ECL1, and S151 in ECL2, differ between humans and rodents [91]. Indeed, binding analyses using human/mouse claudin-5 chimeric mutant (D68E, T75A, and S151T)-expressing cells revealed that the binding of the monoclonal antibodies of clones M48 and 2B12, and clone R2, was attenuated in the D68E and S151T mutants, respectively. Although the binding of clone R9 was partially attenuated in the S151T mutant, the TJ integrity of mouse claudin-5 transfectants was not reduced by R9 due to the insufficient binding affinity of R9 to mouse claudin-5 [87,93]. Thus, these antibodies cannot modulate the TJ barrier function in mice, and their in vivo safety and efficacy have not been clarified.

Although claudin-5 is highly expressed mainly in vascular endothelial cells and functions as a main component of the microvascular endothelial cell barrier [27], claudin-5 also is expressed by epithelial cells in the intestinal tract and lungs [24,25]. However, many other claudin family members and TJ proteins are expressed in these epithelial cells, and the contribution of claudin-5 to the cell barrier in these epithelial cells is unknown. Indeed, anti-claudin-5 antibodies did not affect the barrier function of T84 human intestinal cells [87]. Claudin family member-deficient mice are thought to have no barrier dysfunction in all claudin-expressing tissues for the same reason that they showed a significant lack of barrier function only in certain tissues. When other claudins are able to compensate for claudin-5 function, it is suggested that anti-claudin-5 antibodies can modulate the BBB barrier function without affecting TJ function in other tissues. Currently, our group is conducting experiments in monkeys to evaluate the efficacy and toxicity of an anti-claudin-5 antibody in vivo.

#### 3.1.3. Other Claudin-5 Modulators

Added to the method of directly regulating the function of claudin-5 as described above, it is considered possible to modulate the BBB function by regulating the expression level of claudin-5. Huang et al., showed that polyinosinic-polycytidylic acid [Poly(I:C)], a ligand for Toll-like receptor 3 (TLR3), reduces the expression of claudin-5 in a dose- and time-dependent manner and increases the permeability of the human lung endothelial monolayer [95]. Poly(I:C) induced TLR3-mediated activation of nuclear factor-kappa B (NF-κB), and NF-κB signaling suppressed the transcriptional activity of the claudin-5 promoter [95,96,97]. However, further analysis is needed to determine whether poly(I:C) can control the permeability of the BBB.

Jia et al., reported that high-dose bevacizumab, a neutralizing antibody against VEGF-A, downregulated claudin-5 through upregulation of transforming growth factor β1 (TGFβ1). Conversely, low-dose bevacizumab increased claudin-5 expression via the phosphoinositide 3-kinase (PI3K) pathway [98]. Indeed, VEGF-A downregulated the expression of both claudin-5 and occludin [46,99], and TGFβ1 also decreased claudin-5 expression and increased the BBB permeability in vitro and in vivo [100,101]. Although it is possible to modulate the barrier function with an anti-VEGF antibody, it is difficult to control the expression level of claudin-5 with an anti-VEGF antibody. Therefore, further research is needed for its application to DDS technology for the management of brain diseases.

DDS technology that regulates the BBB permeability by regulating the claudin-5 expression is promising, but further research is required to apply it practically.

### 3.2. Angulin Binders

#### 3.2.1. Fragment of Bacterial Toxins

**Figure 5:**
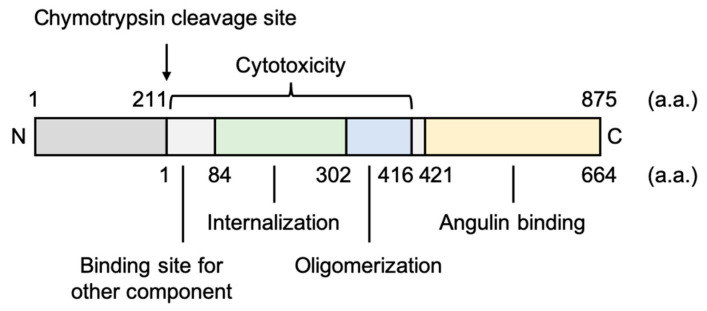
Structure of *Clostridium perfringens* iota-toxin component Ib [102]. The GenBank protein accession number is WP_003463384.

*Clostridium perfringens* iota-toxin is a binary toxin that causes antibiotic-associated enterotoxemia and contains the enzymatic component (Ia) and receptor-binding component (Ib) of adenosine diphosphate (ADP)-ribosyltransferase. Ib is composed of four domains consisting of 664 amino acids (Figure 5) [102]. The three N-terminal domains of Ib play important roles in the organization of the Ib-pore, and the C-terminal domain IV of Ib (Ib421-664; 421–664 amino acids, ~30 kDa), named angubindin-1, binds to its receptors, angulin-1 and -3, but not to angulin-2 [103,104,105]. Angubindin-1 changed the localization of angulin-1 and tricellulin from tTJs to bTJs, thus increasing the permeability of TJs [104].

During an in vitro BBB model, using primary rat brain capillary endothelial cells with rat pericytes and astrocytes, angubindin-1 reduced the TEER value, which was recovered after the removal of angubindin-1 from the culture medium. This finding indicates that angubindin-1 can temporarily modulate the tTJ barrier [90]. Furthermore, in mice, angubindin-1 increased the BBB permeability and was able to deliver a 16-mer gapmer antisense oligonucleotide (5.3 kDa) to the brain. The silencing effect of the antisense oligonucleotide was observed 1–4 h after the injection of angubindin-1. These data indicate that angubindin-1 can reversibly reduce the integrity of the BBB in vivo. Therefore, mice treated with angubindin-1 showed no abnormal behavior and showed normal liver and kidney functions according to histological and serum biochemical tests. Additionally, mice treated with Evans blue solution following angubindin-1 showed no extravasation of Evans blue into the brain [90]. These findings suggest that first-generation angulin binders, such as angubindin-1, can modulate the tTJ barrier and can be safely used for CNS drug delivery in vivo.

#### 3.2.2. Antibodies against Angulin-1

Angulin-1 was originally identified as a lipoprotein receptor (LSR) in the liver [51]. The expression of angulin-1 also correlates with colon, bladder, breast, pancreatic, ovarian, and endometrial cancers [106,107,108,109,110,111]. Recently, Hiramatsu et al., developed the functional monoclonal antibody against the extracellular region of human angulin-1 (anti-hLSR mAb (#1-25)) that crossreacted with mouse LSR. They showed that the antibody inhibited very low density lipoprotein (VLDL)-dependent cell proliferation, and administration of the antibody inhibited tumor growth in mouse xenograft models of hLSR+ epithelial ovarian cancer [110]. They also showed that administration of the anti-hLSR mAb (#1-25) to C57BL/6J mice had no adverse effects on blood or organs. However, there is no information on the effect of this antibody on the BBB function. Further analysis is needed to determine whether second-generation angulin binders, including antibodies, can be a safe drug delivery tool to the brain.

## 4. Conclusions

The BBB plays an important role in protecting the brain from the entry of serum proteins, inflammatory cells, pathogens, and other substances to maintain CNS homeostasis. During the development of safe drug delivery tools to the brain, it is important to modulate reversible and size-selective BBB permeability without BBB disruption. To date, drugs of various sizes, from small molecules to macromolecules, have been developed. Small-molecule drugs have a molecular weight of less than approximately 500 Da; whereas, macromolecules have a wide range of molecular weight, such as in oligonucleotide therapeutics and peptides (7–14 kDa), and antibodies (more than 100 kDa) [112]. Therefore, it is important to control the permeability of the BBB to a suitable size according to the size of each drug. Previously mentioned, the modulation of claudin-5 and angulin-1 enables the BBB permeability of molecules of molecular weight less than 1 and 5.3 kDa, respectively [29,90]. Furthermore, the co-regulation of both claudin-5 and occludin increased the BBB permeability of molecules as large as 4 kDa [46,47]. These findings indicate that targeted TJ components and their combinations are important in controlling the size of molecules that pass through the BBB. 

TJ modulators for drug delivery are classified as first-generation binders, which include toxins and their fragments, and second-generation binders, which include antibodies [69,91]. Shown above, C-CPEs and angubindin-1 are useful tools for studying drug delivery to the brain via the paracellular pathway, but the clinical applications of these molecules are limited due to the immunogenicity. Thus, it is important to develop second-generation TJ binders, such as antibodies and macrocyclic peptides. Owing to the small extracellular region of claudin and its high degree of homology among species, it is difficult to obtain functional antibodies against the extracellular region of claudins [67,68,91,92]. Recently, methods have been developed to obtain functional antibodies against membrane proteins that are difficult to produce, such as claudins (see above) [87,93]. Furthermore, recently, Watari et al., reported a high-throughput screening system based on the time-resolved fluorescence resonance energy transfer method to identify claudin-4 binders. They identified several claudin-4 binders with epithelial-barrier-disrupting activity, such as thiostrepton, using the developed method [113]. These strategies are expected to accelerate the development of new claudin binders that modulate the BBB function in the future. Further development of drug delivery technology to the brain is desired in developing therapeutic agents for CNS diseases, such as Alzheimer’s disease, in the aging society.

## Figures and Tables

**Table 1:** Summary of monoclonal antibodies that recognize the extracellular region of human claudin-5.

Clone	Subtype	Epitope	Binding Specificity ^1^				Effect on TJ Integrity in MDCKII Cells ^2^			Ref
			Human	Mouse	Cynomolgus	Other Claudins	Human	Mouse	Cynomolgus	
M48	Mouse IgG3	2nd loop	+++	-	+++	-	+++	-	+++	[87]
R2	Rat IgG2a	1st loop	+++	-	+++	-	++	-	++	[87]
R9	Rat IgG2b	2nd loop	+++	+	+++	-	+++	-	+++	[87]
2B12	Mouse IgG2a	2nd loop	+++	-	+++	-	++	-	++	[93]

^1^ +++, +, and - indicate strong, weak, and negligible binding reactivity of anti-claudin-5 antibody to claudin-5 in each species. ^2^ +++, ++, and - indicate that the anti-claudin-5 antibodies have strong, normal, and negligible modulating activity on the barrier formation of claudin-5 in each species, respectively.

## Abbreviations

Aβ	Amyloid-β
BBB	Blood-brain barrier
BCEC	Brain capillary endothelial cell
bTJ	Bicellular TJ
C/EBPα	CCAAT/enhancer-binding protein α
CNS	Central nervous system
CPE	Clostridium perfringens enterotoxin
C-CPE	C-terminal region of CPE
DDS	Drug-delivery system
EAE	Experimental autoimmune encephalomyelitis
ECH	Extracellular helix
ECL	Extracellular loop domain
ECS	Extracellular segment
JAM	Junctional adherence molecule
LSR	Lipolysis-stimulated lipoprotein receptor
MARVEL	MAL and related proteins for vesicle trafficking and membrane link
NF-κB	Nuclear factor-kappa B
PDZ	Postsynaptic density-95, discs-large, ZO-1
P-gp	P-glycoprotein
PI3K	Phosphoinositide 3-kinase
Poly(I:C)	Polyinosinic-polycytidylic acid
TAMP	TJ-associated MARVEL protein
TEER	Transepithelial/transendothelial electrical resistance
TGFβ1	Transforming growth factor β1
TJ	Tight junction
TLR3	Toll-like receptor 3
TM	Transmembrane helix
tTJ	Tricellular TJ
VEGF-A	Vascular endothelial growth factor A
VLDL	Very low density lipoprotein
ZO	Zonula occludens

## References

[1] Ehrlich P. (1885). Das Sauerstoff-Bedürfniss des Organismus: Eine Farbenanalytische Studie.

[2] Tsukita S., Furuse M. (2006). The Structure and Function of Claudins, Cell Adhesion Molecules at Tight Junctions. Ann. N. Y. Acad. Sci..

[3] Hashimoto Y., Tachibana K., Krug S.M., Kunisawa J., Fromm M., Kondoh M. (2019). Potential for Tight Junction Protein-Directed Drug Development Using Claudin Binders and Angubindin-1. Int. J. Mol. Sci..

[4] Mikitsh J.L., Chacko A.-M. (2014). Pathways for small molecule delivery to the central nervous system across the blood-brain barrier. Perspect. Med. Chem..

[5] Banks W.A., Greig N.H. (2019). Small molecules as central nervous system therapeutics: Old challenges, new directions, and a philosophic divide. Future Med. Chem..

[6] Hartz A.M.S., Pekcec A., Soldner E.L.B., Zhong Y., Schlichtiger J., Bauer B. (2017). P-gp Protein Expression and Transport Activity in Rodent Seizure Models and Human Epilepsy. Mol. Pharm..

[7] Pardridge W.M. (2005). The blood-brain barrier: Bottleneck in brain drug development. Neurotherapeutics.

[8] Tsukita S., Furuse M., Itoh M. (2001). Multifunctional strands in tight junctions. Nat. Rev. Mol. Cell Biol..

[9] Ikenouchi J., Furuse M., Furuse K., Sasaki H., Tsukita S., Tsukita S. (2005). Tricellulin constitutes a novel barrier at tricellular contacts of epithelial cells. J. Cell Biol..

[10] Abdul Razzak R., Florence G.J., Gunn-Moore F.J. (2019). Approaches to CNS Drug Delivery with a Focus on Transporter-Mediated Transcytosis. Int. J. Mol. Sci..

[11] Rapoport S.I. (2000). Osmotic opening of the blood-brain barrier: Principles, mechanism, and therapeutic applications. Cell. Mol. Neurobiol..

[12] Laksitorini M., Prasasty V.D., Kiptoo P.K., Siahaan T.J. (2014). Pathways and progress in improving drug delivery through the intestinal mucosa and blood-brain barriers. Ther. Deliv.

[13] Masuda S., Oda Y., Sasaki H., Ikenouchi J., Higashi T., Akashi M., Nishi E., Furuse M. (2011). LSR defines cell corners for tricellular tight junction formation in epithelial cells. J. Cell Sci..

[14] Furuse M., Izumi Y., Oda Y., Higashi T., Iwamoto N. (2014). Molecular organization of tricellular tight junctions. Tissue Barriers.

[15] Paris L., Tonutti L., Vannini C., Bazzoni G. (2008). Structural organization of the tight junctions. Biochim. Biophys. Acta.

[16] Furuse M., Fujita K., Hiiragi T., Fujimoto K., Tsukita S. (1998). Claudin-1 and -2: Novel integral membrane proteins localizing at tight junctions with no sequence similarity to occludin. J. Cell Biol..

[17] Mineta K., Yamamoto Y., Yamazaki Y., Tanaka H., Tada Y., Saito K., Tamura A., Igarashi M., Endo T., Takeuchi K. (2011). Predicted expansion of the claudin multigene family. FEBS Lett..

[18] Günzel D., Yu A.S.L. (2013). Claudins and the modulation of tight junction permeability. Physiol. Rev..

[19] Rüffer C., Gerke V. (2004). The C-terminal cytoplasmic tail of claudins 1 and 5 but not its PDZ-binding motif is required for apical localization at epithelial and endothelial tight junctions. Eur. J. Cell Biol..

[20] Furuse M., Sasaki H., Tsukita S. (1999). Manner of interaction of heterogeneous claudin species within and between tight junction strands. J. Cell Biol.

[21] Suzuki H., Tani K., Tamura A., Tsukita S., Fujiyoshi Y. (2015). Model for the architecture of claudin-based paracellular ion channels through tight junctions. J. Mol. Biol..

[22] Ohtsuki S., Sato S., Yamaguchi H., Kamoi M., Asashima T., Terasaki T. (2007). Exogenous expression of claudin-5 induces barrier properties in cultured rat brain capillary endothelial cells. J. Cell. Physiol..

[23] Morita K., Furuse M., Fujimoto K., Tsukita S. (1999). Claudin multigene family encoding four-transmembrane domain protein components of tight junction strands. Proc. Natl. Acad. Sci. USA.

[24] Garcia-Hernandez V., Quiros M., Nusrat A. (2017). Intestinal epithelial claudins: Expression and regulation in homeostasis and inflammation. Ann. N. Y. Acad. Sci..

[25] Kaarteenaho R., Merikallio H., Lehtonen S., Harju T., Soini Y. (2010). Divergent expression of claudin -1, -3, -4, -5 and -7 in developing human lung. Respir. Res..

[26] Morita K., Sasaki H., Furuse M., Tsukita S. (1999). Endothelial claudin: Claudin-5/TMVCF constitutes tight junction strands in endothelial cells. J. Cell Biol..

[27] Kluger M.S., Clark P.R., Tellides G., Gerke V., Pober J.S. (2013). Claudin-5 controls intercellular barriers of human dermal microvascular but not human umbilical vein endothelial cells. Arter. Thromb. Vasc. Biol..

[28] Piontek J., Fritzsche S., Cording J., Richter S., Hartwig J., Walter M., Yu D., Turner J.R., Gehring C., Rahn H.-P. (2011). Elucidating the principles of the molecular organization of heteropolymeric tight junction strands. Cell. Mol. Life Sci..

[29] Nitta T., Hata M., Gotoh S., Seo Y., Sasaki H., Hashimoto N., Furuse M., Tsukita S. (2003). Size-selective loosening of the blood-brain barrier in claudin-5-deficient mice. J. Cell Biol..

[30] Campbell M., Kiang A.-S., Kenna P.F., Kerskens C., Blau C., O’Dwyer L., Tivnan A., Kelly J.A., Brankin B., Farrar G.-J. (2008). RNAi-mediated reversible opening of the blood-brain barrier. J. Gene Med..

[31] Gericke B., Römermann K., Noack A., Noack S., Kronenberg J., Blasig I.E., Löscher W. (2020). A face-to-face comparison of claudin-5 transduced human brain endothelial (hCMEC/D3) cells with porcine brain endothelial cells as blood-brain barrier models for drug transport studies. Fluids Barriers CNS.

[32] Patabendige A., Skinner R.A., Abbott N.J. (2013). Establishment of a simplified in vitro porcine blood-brain barrier model with high transendothelial electrical resistance. Brain Res..

[33] Förster C., Burek M., Romero I.A., Weksler B., Couraud P.-O., Drenckhahn D. (2008). Differential effects of hydrocortisone and TNFalpha on tight junction proteins in an in vitro model of the human blood-brain barrier. J. Physiol..

[34] Berndt P., Winkler L., Cording J., Breitkreuz-Korff O., Rex A., Dithmer S., Rausch V., Blasig R., Richter M., Sporbert A. (2019). Tight junction proteins at the blood-brain barrier: Far more than claudin-5. Cell. Mol. Life Sci..

[35] Uchida Y., Sumiya T., Tachikawa M., Yamakawa T., Murata S., Yagi Y., Sato K., Stephan A., Ito K., Ohtsuki S. (2019). Involvement of Claudin-11 in Disruption of Blood-Brain, -Spinal Cord, and -Arachnoid Barriers in Multiple Sclerosis. Mol. Neurobiol..

[36] Gow A., Southwood C.M., Li J.S., Pariali M., Riordan G.P., Brodie S.E., Danias J., Bronstein J.M., Kachar B., Lazzarini R.A. (1999). CNS myelin and sertoli cell tight junction strands are absent in Osp/claudin-11 null mice. Cell.

[37] Denninger A.R., Breglio A., Maheras K.J., LeDuc G., Cristiglio V., Demé B., Gow A., Kirschner D.A. (2015). Claudin-11 Tight Junctions in Myelin Are a Barrier to Diffusion and Lack Strong Adhesive Properties. Biophys. J..

[38] Castro Dias M., Coisne C., Baden P., Enzmann G., Garrett L., Becker L., Hölter S.M., Hrabě de Angelis M., Deutsch U., German Mouse Clinic Consortium (2019). Claudin-12 is not required for blood-brain barrier tight junction function. Fluids Barriers CNS.

[39] Plain A., Pan W., O’Neill D., Ure M., Beggs M.R., Farhan M., Dimke H., Cordat E., Alexander R.T. (2020). Claudin-12 Knockout Mice Demonstrate Reduced Proximal Tubule Calcium Permeability. Int. J. Mol. Sci..

[40] Ohnishi M., Ochiai H., Matsuoka K., Akagi M., Nakayama Y., Shima A., Uda A., Matsuoka H., Kamishikiryo J., Michihara A. (2017). Claudin domain containing 1 contributing to endothelial cell adhesion decreases in presence of cerebellar hemorrhage. J. Neurosci. Res..

[41] Greene C., Kealy J., Humphries M.M., Gong Y., Hou J., Hudson N., Cassidy L.M., Martiniano R., Shashi V., Hooper S.R. (2018). Dose-dependent expression of claudin-5 is a modifying factor in schizophrenia. Mol. Psychiatry.

[42] Hashimoto Y., Campbell M. (2020). Tight junction modulation at the blood-brain barrier: Current and future perspectives. Biochim. Biophys. Acta Biomembr..

[43] Furuse M., Hirase T., Itoh M., Nagafuchi A., Yonemura S., Tsukita S., Tsukita S. (1993). Occludin: A novel integral membrane protein localizing at tight junctions. J. Cell Biol..

[44] Cording J., Berg J., Käding N., Bellmann C., Tscheik C., Westphal J.K., Milatz S., Günzel D., Wolburg H., Piontek J. (2013). In tight junctions, claudins regulate the interactions between occludin, tricellulin and marvelD3, which, inversely, modulate claudin oligomerization. J. Cell. Sci..

[45] Saitou M., Furuse M., Sasaki H., Schulzke J.D., Fromm M., Takano H., Noda T., Tsukita S. (2000). Complex phenotype of mice lacking occludin, a component of tight junction strands. Mol. Biol. Cell.

[46] Argaw A.T., Gurfein B.T., Zhang Y., Zameer A., John G.R. (2009). VEGF-mediated disruption of endothelial CLN-5 promotes blood-brain barrier breakdown. Proc. Natl. Acad. Sci. USA.

[47] Keaney J., Walsh D.M., O’Malley T., Hudson N., Crosbie D.E., Loftus T., Sheehan F., McDaid J., Humphries M.M., Callanan J.J. (2015). Autoregulated paracellular clearance of amyloid-β across the blood-brain barrier. Sci. Adv..

[48] Krug S.M., Amasheh S., Richter J.F., Milatz S., Günzel D., Westphal J.K., Huber O., Schulzke J.D., Fromm M. (2009). Tricellulin forms a barrier to macromolecules in tricellular tight junctions without affecting ion permeability. Mol. Biol. Cell.

[49] Kamitani T., Sakaguchi H., Tamura A., Miyashita T., Yamazaki Y., Tokumasu R., Inamoto R., Matsubara A., Mori N., Hisa Y. (2015). Deletion of Tricellulin Causes Progressive Hearing Loss Associated with Degeneration of Cochlear Hair Cells. Sci. Rep..

[50] Higashi T., Tokuda S., Kitajiri S., Masuda S., Nakamura H., Oda Y., Furuse M. (2013). Analysis of the “angulin” proteins LSR, ILDR1 and ILDR2--tricellulin recruitment, epithelial barrier function and implication in deafness pathogenesis. J. Cell. Sci..

[51] Yen F.T., Masson M., Clossais-Besnard N., André P., Grosset J.M., Bougueleret L., Dumas J.B., Guerassimenko O., Bihain B.E. (1999). Molecular cloning of a lipolysis-stimulated remnant receptor expressed in the liver. J. Biol. Chem..

[52] Sohet F., Lin C., Munji R.N., Lee S.Y., Ruderisch N., Soung A., Arnold T.D., Derugin N., Vexler Z.S., Yen F.T. (2015). LSR/angulin-1 is a tricellular tight junction protein involved in blood-brain barrier formation. J. Cell Biol..

[53] Daneman R., Zhou L., Agalliu D., Cahoy J.D., Kaushal A., Barres B.A. (2010). The mouse blood-brain barrier transcriptome: A new resource for understanding the development and function of brain endothelial cells. PLoS ONE.

[54] Iwamoto N., Higashi T., Furuse M. (2014). Localization of angulin-1/LSR and tricellulin at tricellular contacts of brain and retinal endothelial cells in vivo. Cell Struct. Funct..

[55] Martìn-Padura I., Lostaglio S., Schneemann M., Williams L., Romano M., Fruscella P., Panzeri C., Stoppacciaro A., Ruco L., Villa A. (1998). Junctional adhesion molecule, a novel member of the immunoglobulin superfamily that distributes at intercellular junctions and modulates monocyte transmigration. J. Cell Biol..

[56] Kostrewa D., Brockhaus M., D’Arcy A., Dale G.E., Nelboeck P., Schmid G., Mueller F., Bazzoni G., Dejana E., Bartfai T. (2001). X-ray structure of junctional adhesion molecule: Structural basis for homophilic adhesion via a novel dimerization motif. EMBO J..

[57] Del Maschio A., De Luigi A., Martin-Padura I., Brockhaus M., Bartfai T., Fruscella P., Adorini L., Martino G., Furlan R., De Simoni M.G. (1999). Leukocyte recruitment in the cerebrospinal fluid of mice with experimental meningitis is inhibited by an antibody to junctional adhesion molecule (JAM). J. Exp. Med..

[58] Yeung D., Manias J.L., Stewart D.J., Nag S. (2008). Decreased junctional adhesion molecule-A expression during blood-brain barrier breakdown. Acta Neuropathol..

[59] Tornavaca O., Chia M., Dufton N., Almagro L.O., Conway D.E., Randi A.M., Schwartz M.A., Matter K., Balda M.S. (2015). ZO-1 controls endothelial adherens junctions, cell-cell tension, angiogenesis, and barrier formation. J. Cell Biol..

[60] Kakogiannos N., Ferrari L., Giampietro C., Scalise A.A., Maderna C., Ravà M., Taddei A., Lampugnani M.G., Pisati F., Malinverno M. (2020). JAM-A Acts via C/EBP-α to Promote Claudin-5 Expression and Enhance Endothelial Barrier Function. Circ. Res..

[61] Laukoetter M.G., Nava P., Lee W.Y., Severson E.A., Capaldo C.T., Babbin B.A., Williams I.R., Koval M., Peatman E., Campbell J.A. (2007). JAM-A regulates permeability and inflammation in the intestine in vivo. J. Exp. Med..

[62] Stevenson B.R., Siliciano J.D., Mooseker M.S., Goodenough D.A. (1986). Identification of ZO-1: A high molecular weight polypeptide associated with the tight junction (zonula occludens) in a variety of epithelia. J. Cell Biol..

[63] Itoh M., Furuse M., Morita K., Kubota K., Saitou M., Tsukita S. (1999). Direct binding of three tight junction-associated MAGUKs, ZO-1, ZO-2, and ZO-3, with the COOH termini of claudins. J. Cell Biol..

[64] Inoko A., Itoh M., Tamura A., Matsuda M., Furuse M., Tsukita S. (2003). Expression and distribution of ZO-3, a tight junction MAGUK protein, in mouse tissues. Genes Cells.

[65] Otani T., Nguyen T.P., Tokuda S., Sugihara K., Sugawara T., Furuse K., Miura T., Ebnet K., Furuse M. (2019). Claudins and JAM-A coordinately regulate tight junction formation and epithelial polarity. J. Cell Biol..

[66] Katsuno T., Umeda K., Matsui T., Hata M., Tamura A., Itoh M., Takeuchi K., Fujimori T., Nabeshima Y., Noda T. (2008). Deficiency of zonula occludens-1 causes embryonic lethal phenotype associated with defected yolk sac angiogenesis and apoptosis of embryonic cells. Mol. Biol. Cell.

[67] Krause G., Winkler L., Piehl C., Blasig I., Piontek J., Müller S.L. (2009). Structure and function of extracellular claudin domains. Ann. N. Y. Acad. Sci..

[68] Evans M.J., von Hahn T., Tscherne D.M., Syder A.J., Panis M., Wölk B., Hatziioannou T., McKeating J.A., Bieniasz P.D., Rice C.M. (2007). Claudin-1 is a hepatitis C virus co-receptor required for a late step in entry. Nature.

[69] Hashimoto Y., Yagi K., Kondoh M. (2017). Roles of the first-generation claudin binder, *Clostridium perfringens* enterotoxin, in the diagnosis and claudin-targeted treatment of epithelium-derived cancers. Pflug. Arch..

[70] Czeczulin J.R., Hanna P.C., McClane B.A. (1993). Cloning, nucleotide sequencing, and expression of the *Clostridium perfringens* enterotoxin gene in Escherichia coli. Infect. Immun..

[71] Hanna P.C., Wieckowski E.U., Mietzner T.A., McClane B.A. (1992). Mapping of functional regions of *Clostridium perfringens* type A enterotoxin. Infect. Immun..

[72] Sonoda N., Furuse M., Sasaki H., Yonemura S., Katahira J., Horiguchi Y., Tsukita S. (1999). *Clostridium perfringens* enterotoxin fragment removes specific claudins from tight junction strands: Evidence for direct involvement of claudins in tight junction barrier. J. Cell Biol..

[73] Katahira J., Inoue N., Horiguchi Y., Matsuda M., Sugimoto N. (1997). Molecular cloning and functional characterization of the receptor for *Clostridium perfringens* enterotoxin. J. Cell Biol..

[74] Suzuki H., Tani K., Fujiyoshi Y. (2017). Crystal structures of claudins: Insights into their intermolecular interactions. Ann. N. Y. Acad. Sci..

[75] Veshnyakova A., Protze J., Rossa J., Blasig I.E., Krause G., Piontek J. (2010). On the interaction of *Clostridium perfringens* enterotoxin with claudins. Toxins.

[76] Freedman J.C., Shrestha A., McClane B.A. (2016). *Clostridium perfringens* Enterotoxin: Action, Genetics, and Translational Applications. Toxins.

[77] Van Itallie C.M., Betts L., Smedley J.G., McClane B.A., Anderson J.M. (2008). Structure of the claudin-binding domain of *Clostridium perfringens* enterotoxin. J. Biol. Chem..

[78] Uchida H., Kondoh M., Hanada T., Takahashi A., Hamakubo T., Yagi K. (2010). A claudin-4 modulator enhances the mucosal absorption of a biologically active peptide. Biochem. Pharm..

[79] Suzuki H., Nishizawa T., Tani K., Yamazaki Y., Tamura A., Ishitani R., Dohmae N., Tsukita S., Nureki O., Fujiyoshi Y. (2014). Crystal structure of a claudin provides insight into the architecture of tight junctions. Science.

[80] Saitoh Y., Suzuki H., Tani K., Nishikawa K., Irie K., Ogura Y., Tamura A., Tsukita S., Fujiyoshi Y. (2015). Tight junctions. Structural insight into tight junction disassembly by *Clostridium perfringens* enterotoxin. Science.

[81] Shinoda T., Shinya N., Ito K., Ohsawa N., Terada T., Hirata K., Kawano Y., Yamamoto M., Kimura-Someya T., Yokoyama S. (2016). Structural basis for disruption of claudin assembly in tight junctions by an enterotoxin. Sci. Rep..

[82] Nakamura S., Irie K., Tanaka H., Nishikawa K., Suzuki H., Saitoh Y., Tamura A., Tsukita S., Fujiyoshi Y. (2019). Morphologic determinant of tight junctions revealed by claudin-3 structures. Nat. Commun..

[83] Vecchio A.J., Stroud R.M. (2019). Claudin-9 structures reveal mechanism for toxin-induced gut barrier breakdown. Proc. Natl. Acad. Sci. USA.

[84] Takahashi A., Saito Y., Kondoh M., Matsushita K., Krug S.M., Suzuki H., Tsujino H., Li X., Aoyama H., Matsuhisa K. (2012). Creation and biochemical analysis of a broad-specific claudin binder. Biomaterials.

[85] Tachibana K., Kondoh M. (2020). A Method to Prepare Claudin-Modulating Recombinant Proteins. Methods Mol. Biol..

[86] Protze J., Eichner M., Piontek A., Dinter S., Rossa J., Blecharz K.G., Vajkoczy P., Piontek J., Krause G. (2015). Directed structural modification of *Clostridium perfringens* enterotoxin to enhance binding to claudin-5. Cell. Mol. Life Sci..

[87] Hashimoto Y., Shirakura K., Okada Y., Takeda H., Endo K., Tamura M., Watari A., Sadamura Y., Sawasaki T., Doi T. (2017). Claudin-5-Binders Enhance Permeation of Solutes across the Blood-Brain Barrier in a Mammalian Model. J. Pharm. Exp. Ther..

[88] Neuhaus W., Piontek A., Protze J., Eichner M., Mahringer A., Subileau E.-A., Lee I.-F.M., Schulzke J.D., Krause G., Piontek J. (2018). Reversible opening of the blood-brain barrier by claudin-5-binding variants of *Clostridium perfringens* enterotoxin’s claudin-binding domain. Biomaterials.

[89] Liao Z., Yang Z., Piontek A., Eichner M., Krause G., Li L., Piontek J., Zhang J. (2016). Specific binding of a mutated fragment of *Clostridium perfringens* enterotoxin to endothelial claudin-5 and its modulation of cerebral vascular permeability. Neuroscience.

[90] Zeniya S., Kuwahara H., Daizo K., Watari A., Kondoh M., Yoshida-Tanaka K., Kaburagi H., Asada K., Nagata T., Nagahama M. (2018). Angubindin-1 opens the blood-brain barrier in vivo for delivery of antisense oligonucleotide to the central nervous system. J. Control. Release.

[91] Hashimoto Y., Yagi K., Kondoh M. (2016). Current progress in a second-generation claudin binder, anti-claudin antibody, for clinical applications. Drug Discov. Today.

[92] Tucker D.F., Sullivan J.T., Mattia K.-A., Fisher C.R., Barnes T., Mabila M.N., Wilf R., Sulli C., Pitts M., Payne R.J. (2018). Isolation of state-dependent monoclonal antibodies against the 12-transmembrane domain glucose transporter 4 using virus-like particles. Proc. Natl. Acad. Sci. USA.

[93] Hashimoto Y., Zhou W., Hamauchi K., Shirakura K., Doi T., Yagi K., Sawasaki T., Okada Y., Kondoh M., Takeda H. (2018). Engineered membrane protein antigens successfully induce antibodies against extracellular regions of claudin-5. Sci. Rep..

[94] Amasheh S., Schmidt T., Mahn M., Florian P., Mankertz J., Tavalali S., Gitter A.H., Schulzke J.-D., Fromm M. (2005). Contribution of claudin-5 to barrier properties in tight junctions of epithelial cells. Cell Tissue Res..

[95] Huang L.-Y., Stuart C., Takeda K., D’Agnillo F., Golding B. (2016). Poly(I:C) Induces Human Lung Endothelial Barrier Dysfunction by Disrupting Tight Junction Expression of Claudin-5. PLoS ONE.

[96] Clark P.R., Kim R.K., Pober J.S., Kluger M.S. (2015). Tumor necrosis factor disrupts claudin-5 endothelial tight junction barriers in two distinct NF-κB-dependent phases. PLoS ONE.

[97] Aslam M., Ahmad N., Srivastava R., Hemmer B. (2012). TNF-alpha induced NFκB signaling and p65 (RelA) overexpression repress Cldn5 promoter in mouse brain endothelial cells. Cytokine.

[98] Jia Y., Qin T., Zhang X., Liu S., Liu Z., Zhang C., Wang J., Li K. (2019). Effect of bevacizumab on the tight junction proteins of vascular endothelial cells. Am. J. Transl. Res..

[99] Laakkonen J.P., Lappalainen J.P., Theelen T.L., Toivanen P.I., Nieminen T., Jauhiainen S., Kaikkonen M.U., Sluimer J.C., Ylä-Herttuala S. (2017). Differential regulation of angiogenic cellular processes and claudin-5 by histamine and VEGF via PI3K-signaling, transcription factor SNAI2 and interleukin-8. Angiogenesis.

[100] Shen W., Li S., Chung S.H., Zhu L., Stayt J., Su T., Couraud P.-O., Romero I.A., Weksler B., Gillies M.C. (2011). Tyrosine phosphorylation of VE-cadherin and claudin-5 is associated with TGF-β1-induced permeability of centrally derived vascular endothelium. Eur. J. Cell Biol..

[101] McMillin M.A., Frampton G.A., Seiwell A.P., Patel N.S., Jacobs A.N., DeMorrow S. (2015). TGFβ1 exacerbates blood-brain barrier permeability in a mouse model of hepatic encephalopathy via upregulation of MMP9 and downregulation of claudin-5. Lab. Investig..

[102] Sakurai J., Nagahama M., Oda M., Tsuge H., Kobayashi K. (2009). *Clostridium perfringens* iota-toxin: Structure and function. Toxins.

[103] Nagahama M., Yamaguchi A., Hagiyama T., Ohkubo N., Kobayashi K., Sakurai J. (2004). Binding and internalization of *Clostridium perfringens* iota-toxin in lipid rafts. Infect. Immun..

[104] Krug S.M., Hayaishi T., Iguchi D., Watari A., Takahashi A., Fromm M., Nagahama M., Takeda H., Okada Y., Sawasaki T. (2017). Angubindin-1, a novel paracellular absorption enhancer acting at the tricellular tight junction. J. Control. Release.

[105] Tachibana K., Kondoh M. (2020). A Method to Prepare a Bioprobe for Regulatory Science of the Drug Delivery System to the Brain: An Angulin Binder to Modulate Tricellular Tight Junction-Seal. Methods Mol. Biol..

[106] García J.M., Peña C., García V., Domínguez G., Muñoz C., Silva J., Millán I., Diaz R., Lorenzo Y., Rodriguez R. (2007). Prognostic value of LISCH7 mRNA in plasma and tumor of colon cancer patients. Clin. Cancer Res..

[107] Herbsleb M., Birkenkamp-Demtroder K., Thykjaer T., Wiuf C., Hein A.-M.K., Orntoft T.F., Dyrskjøt L. (2008). Increased cell motility and invasion upon knockdown of lipolysis stimulated lipoprotein receptor (LSR) in SW780 bladder cancer cells. BMC Med. Genom..

[108] Reaves D.K., Fagan-Solis K.D., Dunphy K., Oliver S.D., Scott D.W., Fleming J.M. (2014). The role of lipolysis stimulated lipoprotein receptor in breast cancer and directing breast cancer cell behavior. PLoS ONE.

[109] Kyuno T., Kyuno D., Kohno T., Konno T., Kikuchi S., Arimoto C., Yamaguchi H., Imamura M., Kimura Y., Kondoh M. (2020). Tricellular tight junction protein LSR/angulin-1 contributes to the epithelial barrier and malignancy in human pancreatic cancer cell line. Histochem. Cell Biol..

[110] Hiramatsu K., Serada S., Enomoto T., Takahashi Y., Nakagawa S., Nojima S., Morimoto A., Matsuzaki S., Yokoyama T., Takahashi T. (2018). LSR Antibody Therapy Inhibits Ovarian Epithelial Tumor Growth by Inhibiting Lipid Uptake. Cancer Res..

[111] Shimada H., Satohisa S., Kohno T., Takahashi S., Hatakeyama T., Konno T., Tsujiwaki M., Saito T., Kojima T. (2016). The roles of tricellular tight junction protein lipolysis-stimulated lipoprotein receptor in malignancy of human endometrial cancer cells. Oncotarget.

[112] Shen X., Corey D.R. (2018). Chemistry, mechanism and clinical status of antisense oligonucleotides and duplex RNAs. Nucleic Acids Res..

[113] Watari A., Kodaka M., Matsuhisa K., Sakamoto Y., Hisaie K., Kawashita N., Takagi T., Yamagishi Y., Suzuki H., Tsujino H. (2017). Identification of claudin-4 binder that attenuates tight junction barrier function by TR-FRET-based screening assay. Sci. Rep..

